# Using a community geography place-based approach to explore the impact of a regional research infrastructure in England

**DOI:** 10.1186/s12961-025-01380-2

**Published:** 2025-10-16

**Authors:** Bryony Porter, Claire Thompson, Wendy Wills

**Affiliations:** 1 National Institute for Health and Care Research Applied Research Collaboration East of England, Douglas House, Cambridge, CB2 8AH England; 2 Cambridgeshire and Peterborough Foundation Trust, Cambridgeshire, United Kingdom; 3 Centre for Research in Public Health and Community Care, School of Health & Social Work, University of Hertfordshire, Hatfield, Hertfordshire, AL10 9AB United Kingdom

**Keywords:** Place-based, Research infrastructure, Contributions analysis, Underserved communities, Health inequalities

## Abstract

**Background:**

There is a need to critically examine both how research infrastructures interact with the populations they serve and the perceived effects of these interactions. This paper reports on a contribution analysis-informed study of a research infrastructure and its place-based approach to working with local communities – the National Institute for Health and Care Research Applied Research Collaboration East of England (NIHR ARC EoE). The aims were (1) to understand the perceived impact of the NIHR ARC EoE place-based approach and (2) to explore its processes and challenges.

**Methods:**

From April–June 2023, we interviewed 11 research staff from the infrastructure (NIHR ARC EoE) and nine community-based partners who had worked with NIHR ARC EoE since 2019. The interviews explored experiences of developing research partnerships, learnings, outcomes and challenges. The interviews were audio-recorded, transcribed and subject to a thematic analysis. The findings were subsequently mapped onto a Research Contributions Framework.

**Results:**

The place-based approach was characterized as relationships-driven and community-focused in building research infrastructure, which improved motivation and commitment to local involvement in research. Three perceived impacts were highlighted: working with underserved communities, cross-sector relationship development and building skills and research capacity. Key barriers included differing expectations of research timescales, a fear of problematizing communities, and intensive resource requirements for developing foundational level relationships.

**Conclusions:**

The place-based approach enabled opportunities to work with (rather than do to) communities previously underserved by research and where the development of trusting relationships was key. However, strategic efforts to dismantle bureaucratic barriers must be developed to maximize reach and potential. The findings present an effective approach to understanding the impact of a place-based approach to working with communities. The value of a place-based approach is widely applicable to any research infrastructure aiming to collaborate, involve and engage communities in research.

## Background

### Research infrastructure

Health and social care services research does not occur in a vacuum. It is necessary to look beyond the pipeline of research and the interaction between the funders of projects and the teams of researchers who deliver them. Research is supported and enabled by complex and sometimes overlapping systems of research infrastructure (RI). Research infrastructures are defined as “facilities that provide resources and services for the research communities to conduct research and foster innovation in their fields” [1]. Research infrastructures are varied in their remit, implementation and size, spanning from large facilities, specialist equipment and collaboration networks to e-infrastructure networks, collections and libraries. Research infrastructures exist across research disciplines and are used across the research lifecycle. Often this includes substantial national or international investment [2] in facilitates for early stage experimental science (equipment, laboratories and collaborations), biomedical clinical research facilities [3] and collaborations. Investment also occurs in institutions (e.g. universities) to support researchers in specialist areas (e.g. mental health, cancer, genetics and developmental medicine) through to applied health and social care research. Infrastructures have also been set up to support the delivery of research (e.g. recruitment of participants into studies) [4, 5]; invest in partnerships across health and social care institutions, universities and communities to support the translation of research into practice [6]; and increase research skills and expertise in the workforce [7]. They can be located in one place or across multiple sites acting as hubs or platforms [8] that can be key for collaboration within regions, nationally and internationally [9].

Large investments of public money have facilitated the establishment of research infrastructures on regional, national, European and international levels, across a multitude of disciplines and the entirety of the research pathway [4]. One of the major funders of health and social care research in the United Kingdom is the National Institute for Health and Care Research (NIHR), funded by the United Kingdom Government Department of Health and Social Care. The NIHR fund several research infrastructures and have invested more than £606 million each year in research infrastructure for services, facilities and people to support research and its delivery [10]. A key priority of NIHR is to build diverse, inclusive and impactful public partnerships through the involvement and engagement of patients, service-users, carers and the public.

Engaging communities in research through the development of meaningful public partnerships is increasingly prioritized by healthcare systems, funders and local communities and organizations [11, 12]. There is a spectrum of inter-related and overlapping approaches, such as community-based participatory research (CBPR), community engagement, patient and public involvement, service user engagement, stakeholder engagement, participatory research and participatory action research (PAR) [13]. Furthermore, recent years have seen a substantial drive to improve inclusion of underrepresented communities in clinical and applied research, including communities and regions with high health needs and coastal, rural and semi-rural areas with large ageing demographics [13].

The involvement of communities across all stages of research can enhance the quality and appropriateness of research [14] and increase knowledge, skills and confidence among individuals involved [15]. However, there is no standardized way of operationalising or assessing diverse, inclusive and impactful public partnerships and public involvement within research infrastructures [8, 16, 17]. This is especially challenging owing to the many potential influences and forms of impact across multiple public partnerships, stakeholders and systems, which are difficult to measure and quantify [18]. Added to which, expectations of impact vary from the different perspectives of stakeholders [19]. In this paper, we focus on one infrastructure system, the NIHR Applied Research Collaboration East of England (NIHR ARC EoE) and explore how its approach to working with underserved communities might be conceptualized and assessed.

### Applied research collaborations (ARC)

Applied health and care research typically aims to provide practical benefits to patients, the public, and health and social care staff through improvements in health and care services and delivery [20]. NIHR Applied Research Collaborations are part of a sustained investment in applied health and care research and build on a previous iteration of regional infrastructure, the Collaborations for Leadership in Applied Health Research and Care [7]. The aim of NIHR Applied Research Collaborations (ARCs) is to “undertake high-quality applied health, public health and social care research with a focus on generalizable learning at a regional and national level. Working closely with stakeholders, including the Integrated Care Systems (ICSs), the Health Innovation Networks (HINs) and other NIHR research infrastructure, the ARCs will also support knowledge mobilization and implementation of research-based evidence to ensure effective interventions and models of care can be scaled nationally, thereby maximizing the impact of research”. [21].

The NIHR ARC EoE is 1 of 15 ARCs across England, as part of a £135 million investment [22] (from 2019 to 2024) to support applied health and care research that responds to, and meets, the needs of local populations and local health and care systems. The NIHR ARC platform takes a regional approach and places a heavy influence on meeting the needs of specific populations and areas. The NIHR describes this approach as funding “local collaborations (regional ARCs) to support applied health and care research”. [23] To date, NIHR ARC EoE has worked with over 200 collaborating organizations since 2019, across a multitude of research engagement and research projects. The collaboration has seven research themes, encompassing population health and care across the life course, engaging with underserved communities, addressing health inequalities and applied health and social care research priorities. As part of the funding allocation, NIHR ARC EoE committed to improving engagement and involvement of communities in the region that had previously been underserved by research. The overall aim is to facilitate deep and sustainable engagement with communities over time and across the region.

### Community geography: a place-based approach

This place-based approach can be understood as a long-term and strategic effort of the research infrastructure to work collaboratively with local health, care and charity organizations and people in underserved communities. In doing so, research teams are seeking to research with, rather than research on, local areas and groups. Such an approach has been underpinned by fact-finding and relationship-building; collaborative learning; and informed, wide-reaching engagement [24]. It is grounded in the assumption that local engagement enhances the quality of the research produced [25].

**Fig. 1:**
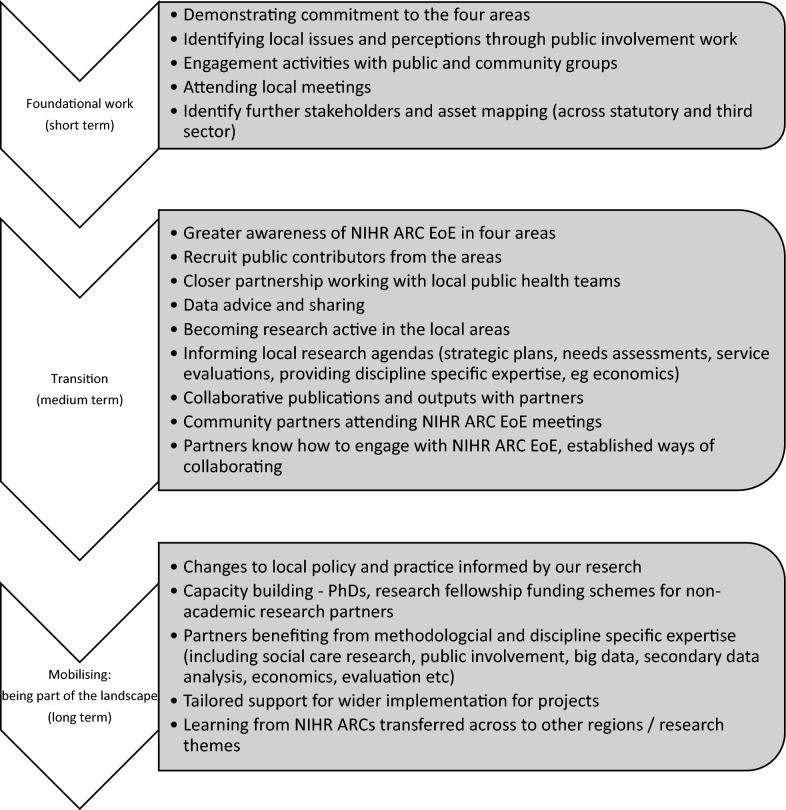
Stages of developing a place-based approach through research infrastructure

Across NIHR ARC EoE infrastructure, all research themes engage locally, and a variety of approaches to engaging with the four identified areas have been taken – some following a graduated strategic approach, focusing on short-, medium- and long-term goals, whilst others have been more ad hoc and opportunistic. Generally, this has translated into three broad stages: foundational work; transition; and mobilization (Fig. 1).

In terms of theorizing our approach, the region-specific nature of the place-based approach to working with underserved communities, as described above, closely aligns with the principles of community geography. Specifically, the notion that community is usually associated with social ties, interactions and an expression of the common good and the ethics of care [27] has significant resonance with the underlying principles of collaborative local public health research. Community geography, a framework originating in North American universities with a long tradition of public engagement [28], represents a small but growing subfield in geography and applies social science methodologies to community problems, with a particular focus on the impacts of inequalities [29]. Complex, real-world problems (especially around health) do not reside in the academy alone, but rather at the intersections of sectors, disciplines, programs, cultures, communities and people [30].

Community geography takes a pragmatic approach enacted by local alliances of academic researchers, policy-makers, residents, charities and sometimes activists. These alliances focus on work that enables underserved communities to address local challenges (such as health and social inequalities) [26]. Building reciprocal relationships is central to achieving longer-term impacts. Creating research of public value requires a deep understanding of local needs, demands and preferences [27]. The approach positions community as emergent from a process of social inquiry, rather than denoting a pre-existing public awaiting engagement [28]. It involves an explicit emphasis on identifying the already existing knowledge base emerging from local residents’ lived experiences and facilitates place-based approaches to identifying and solving community-based issues [29].

The place-based approach is intended to address the challenges of reaching underserved communities who face reduced opportunities to engage with and influence research and services because of structural barriers. This is a pertinent issue for rural communities that may have reduced receptivity for research and capacity to engage because of issues such as travel costs, time, lack of familiarity with research processes, lack of community resources and gatekeepers to facilitate, and a failure by researchers to study locally relevant problems and contexts [13]. Overcoming such challenges requires longer-term and more stable ties to partners and communities outside of the academic environment that are informed by local context and can also help counteract redundant or potentially exploitative practices of engagement that prevailing systems of research can inadvertently produce [30]. Building longer-term ties and close collaborations in this way positions universities as anchor-institutions that serve as a stabilizing force and key actors in the development and maintenance of communities. From a geographical perspective, they can be understood as crucial resources for improving their host communities by engaging with and supporting them. While this theoretical perspective provides an apt justification for our approach, it does not bring us any closer to understanding and measuring its impacts in a way that is congruent with public health concerns and audiences. For this, we turn to the Research Contributions Framework.

### Assessing our work with communities: a Research Contributions Framework (RCF)

It is notably difficult to demonstrate the value of research infrastructures and the multifaceted influences they entail against the backdrop of differing expectations of impact [18, 19]. There is an increasingly present demand for evidence of the impact of investment in research infrastructure. Traditional popular methods of assessing impact have focused on cost–benefit analysis or analysis of the relevant research outputs of research infrastructure (e.g. publications and citation numbers, number of researchers trained). However, these approaches do not account for the complexity of research infrastructure and the wide potential reach of investment through research, resources, collaborations, connections, people and within existing established research active organizations (e.g. universities, healthcare and charities) and connecting with diverse communities for engagement and involvement in shaping and participating in research [8]. This highlights the need to explore alternative approaches that can holistically account for the impact of investment in research infrastructure.

The Research Contributions Framework (RCF) [31] provides one way of exploring the impact of our place-based approach and assessing the impact of research infrastructure. The RCF is an empirical framework for assessing research impact and is based on contribution analysis [31, 32]. It acknowledges the complex systems and environments in which research operates [31], and is particularly useful for exploring the indirect aspects of research impact, such as influencing and upskilling of the public and research-users, and building relationships and networks of research users [33]. The Research Contributions Framework has been used to assess the impact of patient and public involvement in health research studies accounting for contributions to study design and tailoring of research to address key patient needs and unexpected benefits, such as peer support and a sense of purpose for public contributors [34]. Zakaria and colleagues argued for a contribution analysis approach to be applied to research infrastructures to articulate the unique aspects and benefits of infrastructure [8]. The approach is being applied here to consider how the influence of engagement with communities through research infrastructure is incorporated into existing community knowledge, systems, understanding, beliefs and experiences. This aligns with concerns over the complicated process of doing community geography and the need to critically reflect upon its interactions and outcomes [35]. In this paper, we explore whether the RCF can be applied to assess the impact of our place-based approach.

This paper presents the findings from our review of the NIHR ARC EoE place-based approach working with four diverse local areas. The aims were to (1) understand the perceived impact of the place-based approach and (2) to explore the processes and challenges associated.

## Methods

Data was collected between April and June 2023. All data were collected by the first author. Overall, 11 NIHR ARC EoE research staff and nine community-based partners from the four areas participated in the study.

### Context

**Fig. 2:**
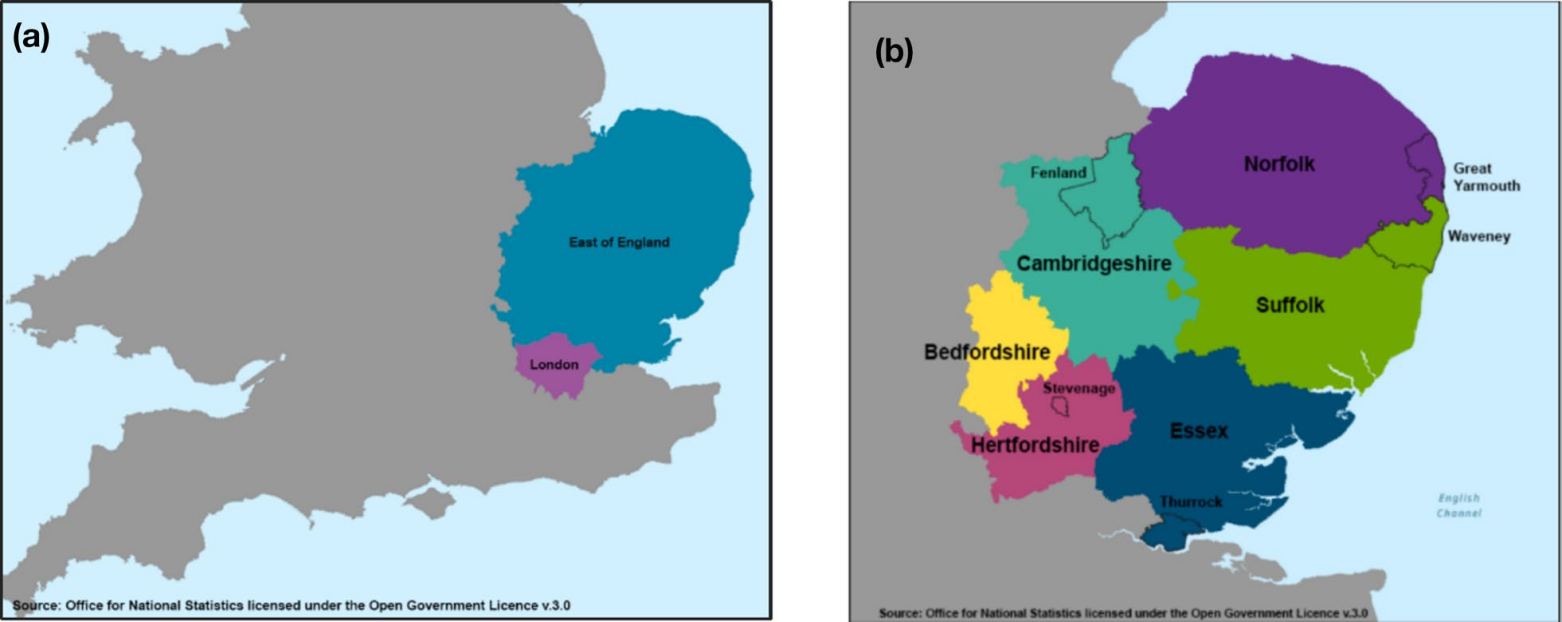
(**a and b**) The East of England and the four main areas for engagement

The East of England is home to 6.3 million people [36] across the counties of Bedfordshire, Cambridgeshire, Essex, Hertfordshire, Norfolk and Suffolk (Fig. 2a and b). There are rural, urban and coastal communities and variation across areas of significant affluence and deprivation, as well as coastal communities with poor health outcomes [37]. NIHR ARC EoE is a local collaboration, bringing together providers of health and social care services in the region, including six National Health Service Integrated Care Systems, four universities in the East of England (Hertfordshire, East Anglia, Essex and Cambridge), local authorities, charities and community organizations, and an organization specializing in implementation of research into practice across the region. In part, this is achieved by taking a place-based approach and focusing efforts on four areas in the region that are characterized by relatively high levels of deprivation, health inequalities and poor outcomes [38]. They also have been underserved by the research community and have had few opportunities to participate in or shape research. The four areas cover a collective population of around 800 000 people and are: (1) the coastal and rural areas of Waveney and Great Yarmouth, where 31% live in areas among the 20% most-deprived in England [39]; (2) Stevenage, a town in Hertfordshire with 16% of children living in low-income families [40]; (3) Peterborough, a diverse and socioeconomically deprived city [41], along with Fenland [42], a rural, largely agricultural area, where the health of the population is generally worse than average; and (4) Thurrock, a Thames Gateway so-called commuter town area immediately east of London, with an ethnically diverse population and high levels of health behaviour risk factors (smoking, physical inactivity and overweight) [43] (Fig. 2a and b).

While the four areas share certain characteristics and profiles (as explained above), they were purposively selected for their diversity in terms of location and population. Collectively, they are diverse in terms of ethnicity, ageing populations, and isolated and socioeconomically disadvantaged communities. Focus on these areas was a deliberate attempt to help inform measures to mitigate and address health inequalities across the region and establish meaningful and sustainable engagement with these communities.

### Recruitment

Initially, we recruited the researcher sample sending email invitations to each of the seven research theme leads to take part in an interview or to nominate up to two representatives to be interviewed. We also invited a representative from the research implementation workstream to be interviewed. A total of 13 were approached, and 11 agreed to be interviewed. We then used a snowballing technique to recruit community partners nominated by the researchers: individuals with an established relationship and those who had been involved with NIHR ARC EoE projects. We purposively sampled at least two community partners from each of the four local areas: Great Yarmouth and Waveney, Stevenage, Thurrock, Peterborough and Fenland, and ensured that different types of community-based partners were sampled (e.g. health and social care, local authority, charity and public contributor). All participants were researchers or community partners from the East of England.

### Data collection

All participants were invited to take part in a semi-structured interview with the first author, either in-person or virtually. Interviews lasted between 30 min and 2 h. The topic guide explored experience of developing relationships and projects, understanding and perceived impact of a place-based approach, and barriers and facilitators to successful partnership working. We also asked about views of the place-based approach, what helps to sustain relationships and what could be improved.

### Data analysis

Interviews were transcribed electronically and anonymized. The transcripts were analysed inductively using thematic analysis approach [44]. Data were analysed and interpreted as they were available, allowing for an iterative approach to the identification and development of themes. The first author analysed all transcripts, and the third author independently analysed a random subsample of four transcripts (20%). Themes were discussed and refined within the research team as an iterative process. Direct (anonymized) quotations are used to illustrate key themes. Data management utilized Nvivo12. The interview findings were used alongside existing reported information from projects in the four areas (Great Yarmouth and Waveney, Thurrock, Stevenage and Peterborough) to map onto the Research Contributions Framework.

### Positionality

All authors identify as white women with extensive experience in qualitative, applied health and public health research. We were involved in this study as members of the regional research infrastructure and received funding through our roles within it. As such, we approached this assessment from the standpoint of academic insiders, with a shared commitment to critically reflecting on our positionality throughout the research process.

The first author had prior working relationships with some participants within the infrastructure, although many, including all community partners, had no existing connection with the interviewer. This dual positioning as both familiar and unfamiliar to participants may have influenced the dynamics of the interviews and the data shared. Furthermore, community partners were invited to participate on the basis of their involvement with the infrastructure, which may have led to a sample skewed towards those with more positive experiences. We acknowledge the possibility that individuals with less favourable or more limited engagement may have been underrepresented.

We recognize that our roles within the infrastructure may have shaped our interpretations and interactions, and we have sought to remain reflexive and transparent about these influences throughout the research.

### Ethics

Informed consent was obtained from all participants prior to interview, and permission was sought to audio-record the interviews. Ethics approval was granted by University of Hertfordshire Health, Science, Engineering and Technology ECDA HSK/SF/UH/05295.

## Results

From April to June 2023, we interviewed 11 research staff from the infrastructure (NIHR ARC EoE) and nine community-based partners who had worked with NIHR ARC EoE since 2019. Research staff were all researchers or academics who were employed at one of the partner universities or organizations of the research infrastructure. Four researchers worked at the University of East Anglia, four at the University of Hertfordshire and two at the University of Cambridge. Seven researchers were senior academics who also held leadership positions in the research infrastructure as research theme leaders.

Community partners worked or lived in the four areas, including three from Great Yarmouth, two from Stevenage, two from Peterborough and Fenland, and two from Thurrock (see Fig. 2 for locations). One community partner was a public contributor who was involved with one of the research themes. The remaining worked in across community and charity organizations (*n* = 3), in health and social care (*n* = 3) and local authorities (*n* = 2).

Overall, the community geography place-based approach was described by participants as valuable, motivational and demonstrating commitment to local communities. Three areas of perceived impact emerged from the analysis: working with underserved communities, cross-sector relationships and building skills and research capacity. However, the approach was notwithstanding its challenges, including differing expectations of project timescales in academia compared with public sector organizations, a fear of problematizing communities by identifying them as “in need” and intensive resource requirements for developing foundational level relationships.

### What did the community geography place-based approach achieve?

There was an individuality in perception, interpretation and interaction with the place-based approach and therefore what the perceived impacts are. Moreover, there was a varied interpretation from individuals, groups, organizations and communities that research infrastructures aim to engage and involve. Despite these nuances, there were three areas of achievement particularly highlighted in our review: working with underserved communities, cross-sector relationships and building skills and research capacity.

### Working with underserved communities in research

The benefit of being funded for 5 years through the research infrastructure provided an extended period to develop relationships with communities and better understand the local systems and dynamics within communities. Importantly, the funding period provided time to implement the stages of developing a place-based approach through research infrastructure (Fig. 1).

“I think that’s what’s quite useful in some ways about ARC where you get an extended period of time, 4 or 5 years, so people aren’t going anywhere for a little while and then you can have regular things…so there’s a little bit of familiarity and structure that people feel they can hang things on instead of you just…not harassing or just bothering people, whereas if there’s something social going on it’s a bit nicer for people I think” (NIHR ARC EoE Researcher (4)).

Despite lengthy lead-in time and negotiations through bureaucratic barriers, research projects with underserved communities did come to fruition. Critical to this was trusting relationships with communities and key members of communities in local areas. Some relationships were built on pre-existing connections and networks from the researchers’ own networks and from the legacy of previous iterations of research infrastructure funding in the region. However, many others were developed as a result of the research infrastructure priorities for engaging with specific, diverse areas in the region, prioritising focus in particular areas and enabling through research infrastructure resource and funded time to develop relationships. This went some way to overcoming the inherent precarity at the intersection of community movements and specific project-funded researchers [45]. In Peterborough, NIHR ARC EoE researchers actively involved members of the Muslim community to tailor their approach to data collection and to collaboratively decide how to discuss a bowel cancer screening intervention in a culturally appropriate way. As part of an evaluation of The British Islamic Medical Association (BIMA) intervention for bowel cancer screening in the Muslim community, building relationships first was particularly important in communities where trusted individuals were the key link into working with the community. Collaboration across a network to link with underserved communities helped to ease the burden of repeated interactions of those communities with research or statutory organizations.

“That for me was like gold, is it called the gold dust? When you get that kind of information that is very difficult for you to kind of get otherwise because this community, they are kind of very…they don’t trust statutory services. So to me it was like a paved road that was there and [researcher] opened it for us, and then I could hear and talk about with them what they [the community] think is important” (Community Partner, Local Authority).

### Established cross-sector working relationships

Mutually beneficial and productive working relationships were established between research and health and social care, local authorities and charity organizations. Cross-sector working brought together the strengths of different areas of expertise that paved the foundation for close and functional working relationships that will last beyond the lifetime of NIHR ARC EoE. This included collaboration on research funding applications, subsequent funding and research projects that emerged as part of the working relationships that were developed, highlighting the potential role of research infrastructure engagement in influencing existing community knowledge and systems.

“So I think the biggest outcome was just the relationship everyone built together. It really felt like we had created a bit of a sort of network. So to this day I still have a really good personal relationship with [local government/Council] on the back of doing that work and I don’t really have that strong a relationship with any of the other local Councils, but because we did that work with the Council, then had a close relationship with the leads there, to this day we still have a good relationship there as well as with the University and [community organization] as well. So, building that network I think was a great outcome” (Community Partner, Patient Advocacy Organization).

In Thurrock, researchers spent time talking and listening to those living and working in the local area, to understand local issues and particular health and social care priorities. Informal conversations subsequently led to more established relationships in the area, attending local meetings and visiting existing local groups. Connections in the local area were invited to research theme meetings for shared learning and to develop research priorities. The reciprocal relationship was integral and helped to form the foundation for networks for collaboration and working with underserved communities, to develop community-driven research projects. This has included a series of funded projects including, for example, working with Gypsy, Roma and Traveller communities in Essex, in collaboration with members of the community, charities, local government and health and social care systems. The long-term, foundation building work is particularly important here in working with communities where individual trusted relationships are fundamental.

### Building skills and research capacity

A key role of research infrastructure is the development of research capacity and a skilled workforce, and the place-based approach encouraged community-focused research priorities for researchers. In addition, it encouraged building skills and knowledge of research in local groups, organizations and communities, as well as a mutually beneficial supportive space for sharing lived experience with others and incorporating the engagement into their existing community knowledge and experiences.

“So it’s actually become quite important to them to be part of a research group and then they see the reports and they’re building as a group as well. So you know they’re meeting each other and going through experiences between them. So it’s been really positive on several levels, but particularly, I would say for the group members to feel part of something that that they’ve got something to say that people are listening to” (Community Partner, Community and Voluntary Organization Lead).

For both researchers and community partners, confidence and experience in applying creative and flexible approaches to public involvement in research was increased.

“It wasn’t anything I had tried before, so it was a really good learning experience on my behalf and it was really good to work with a whole different range of people as well…It was a whole different range and mix of people bringing all different skills and experience, so it was really valuable, and I think everyone felt that they had learnt so much” (Community Partner, Patient Advocacy Organization).

For some, the requirement to work in certain areas also expanded their critical reflection of variation in the experience of communities in different areas (e.g. how might food poverty impact coastal or rural communities differently). This is an indirect benefit of the approach through the mobilization of knowledge and sharing of experience and expertise across sectors.

“It’s a learning curve, you find out about the places and what’s going on there as you go along. It’s definitely changed the way I approach my work…But speaking to people in them…it kind of forces you to look at it through that way, so yeah, it’s been useful” (NIHR ARC EoE Researcher (04)).

The infrastructure provided links to a wider network and for some researchers, provided a launching point for career progression and the opportunity to have their time (usually funded through universities) bought-out for dedicated time to be involved in research-related activity. This included dedicated time for developing relationships with communities for projects and “time to facilitate an NIHR application” (NIHR ARC EoE Researcher (01)). At points, small pockets of funding were also available from NIHR ARC EoE, specifically for public involvement or developing work in the four local areas.

“But from NIHR and ARC they talked about leadership development, so now I’m part of the NIHR Leadership Academy, and also about building capacity. So, what we’ve got now is that all of that is again attributable to ARC funding, undeniably” (NIHR ARC EoE Researcher (01)).

### What were the practical challenges?

### Establishing commitment and shared understanding

All participants were in favour of the focus on local level-engagement. However, during the interviews it was apparent that there was not a complete and shared understanding of the approach itself. Depending on the sector and role, different stakeholders were responsible and invested in differing aspects of the approach. Most important to community partners who were interviewed was that the approach was localized and that there were underpinning values in how communities were engaged and involved. These values are inherent in the achievements of this approach and the network of individual connections and relationships developed across research infrastructure.

“We’re [local healthcare provider] very much championing that sort of placed-based approach to reducing inequalities and therefore I think that that or the other work you do around it, like engagement, co-production needs to model that approach, so I think it’s good to have that local research” (Community Partner, NHS Integrated Care Board).

The place-based approach aimed to directly engage people and communities that have previously been underserved by research, meaning that more effort was required to develop a baseline level of understanding and interest in the research process. Preconceptions of research can be a considerable barrier to involvement, particularly when working with community partners that are unfamiliar with the research process. Greater care was needed to ensure that the engagement was accessible, comfortable and welcoming, and acknowledging the value of contribution and involvement. This included being flexible in the method or approach used, “meeting people where they are” in terms of their previous experience and preconceptions of research, and in terms of the physical space that is familiar and comfortable. For example, the Stevenage Dementia Involvement Group takes place in The Red Shed, a community garden and space for people with dementia, helping to reduce initial barriers to involvement by being in a space that is familiar and comfortable to the group already.

“So, I think that’s quite important, specifically around dementia is to meet people somewhere where they’re used to within their own community” (Community Partner, Community and Voluntary Organization Lead).

Having a clear understanding of involvement, and managing expectations about what could be achieved, enabled good working relationships to develop. Sometimes, limited expectations of the outcome of engagement were beneficial, creating space to build relationships and develop ideas from the ground up. However, others noted the benefit of approaching community partners with something tangible and evidence-based, providing a clear message and something to work with and from.

“Sometimes people are doing this on top of their business as usual, they’re doing it because they really think they believe in it or they really just want to and I think you’ve got to respect the fact that some people are doing this and it is an above and beyond and that we all should be showing each other respect and thanks for the effort that some people are putting in” (NIHR ARC EoE Researcher (02)).

“I think because we weren’t very clear, you know, I’ll take that on board as well, we weren’t very clear, about what and how we wanted to collaborate and actively take it forward, I think that might have been why it didn’t go or it didn’t get through for us…I think it’s just about being, I suppose being clear about what, you know, what’s needed and timelines and things because I know that they change and then people’s capacity to do it changes and funding may not appear in the same way” (Community Partner, Local Authority).

### Maintaining relationships

Building and sustaining relationships within specific areas required significant time, effort and resource. Considerable foundational work was often required, including one-to-one meetings, team meetings, working groups, presentations, phone calls, follow-up meetings and then continued regular meetings or communication.

“I think one of the biggest challenges is especially there are particular key people a lot of times in those regions, and first of all, finding those people, and then developing and sustaining good relationships with those individuals are all very challenging” (NIHR ARC EoE Researcher (06)).

Consistent communication was valued, particularly for public sector organizations who may find the relatively slow pace of research processes frustrating and at odds with organizational (and community) demands.

“Some of the challenge is probably time, capacity and also research, academia, it moves a lot slower isn’t it, and there’s not that much urgency apart from when the deadline comes, but whereas for us it’s like every quarter we do a report to the regional team and maybe two or three quarters of quarterly reports have gone forward where they might just say [underserved population] group, we are participating in the University research you know, we don’t have much to say…I guess it just is what it is because it’s one of these programmes that sometimes you just need to take your time, but I do think a lot of that time is wrapped up in bureaucracy probably, academic bureaucracy” (Community Partner, NHS Integrated Care Board).

The foundational work (Fig. 1), through initial meetings, conversations and mutual priorities, helped to build trusting relationships. The relationship should be mutually beneficial, whether this equated to something that was of benefit for the organization, work supporting their priority areas, reimbursement or resources. Failure to follow-up on contacts initiated by NIHR ARC EoE negatively impacted community partners’ perceptions of being involved in research.

“Sometimes people have asked for some advice or asked me to make contact and I have and then they haven’t always come back to me and that I find a little, well, not soul destroying, but a little bit difficult because you know you make a point of asking me for my contact details and ask me where I can be involved and then you don’t even acknowledge it, you know? So that annoys me” (Community Partner, Public Contributor).

“I think one of the best ways to build trust with organizations in the third sector is to really demonstrate your appreciation for them, an acknowledgement about what they do and help them with their work. Not your work. Not our work but help them” (NIHR ARC EoE Researcher (07)).

### Operationalizing collaboration

Operationalizing meaningful local-level working was not without its challenges. Researchers described being encouraged to inclusively represent experiences of communities but were unsure – especially in the early stages of ARC – how to move from having interesting conversations with individual people (foundational work) to feeding into local research agendas and strategy (mobilizing). In other words, they were conscious of “how to include the community level of experience and activity and not just simply reducing it down to the people who we happen to talk to individually” (NIHR ARC EoE Researcher (12)). Some researchers described how, at the start, the approach felt “disconnected from the people on the ground” (NIHR ARC EoE Researcher (09)), particularly in areas without pre-established relationships, but that this had eased over time with examples of community-driven projects. Further, the localized approach sometimes conflicted with the national and international research impact agendas of universities. Others noted that involvement with projects within the four areas had been relatively coincidental, rather than planned.

“I mean, for me it [research area] was of interest anyway because of its content, but also because it was sitting within one of our [identified areas] which also just made it useful given, as I said before, the specific areas have not been present in my work…But the area has not been a driving presence in the project, so it just doesn’t make sense to talk about it as a place-based related project, even though we can probably pinpoint to areas within the [four areas], if you know what I mean” (NIHR ARC EoE Researcher (09)).

For some, identifying communities as deprived or in need was uncomfortable and, although well-meaning, could be perceived negatively and at times felt disconnected from communities (although this had improved over time). The researchers were conscious of who was being left out in the decision to focus on particular areas, how this might widen inequalities, and that there was an uneven distribution of effort and engagement across the areas.

“I don’t completely agree with identifying communities as deprived, in need and all of that kind of language that go with it” (NIHR ARC EoE Researcher (01)).

While identifying and targeting efforts within certain areas might make sense from an academic or policy perspective, the rationale could have negative connotations on the ground, including stigma and a focus on deficits rather than assets.

A challenge of involvement with the research infrastructure was the high level of bureaucracy required to involve a variety of stakeholders, such as the extensive requirements for identification to reimburse public contributors for their involvement, and funder reporting burden, and overly rigid cofunding or memorandum of agreements.

“Is there anything we could do not to scare people off with the co-funding or the memorandum of agreement where you have to put like a monopoly money figure on something…I do get the rationale for why the NIHR want to do that, but it feels very heavy to some groups… I suppose it feels a bit untrusting or extractive when it’s ‘Yeah, can you sign this form? Can you turn up for this thing? Can you fill in this agreement?’ That doesn’t feel very community” (NIHR ARC EoE Researcher (04)).

While the emphasis was on inclusive and cross-sector collaboration, the logistics and detail of achieving this were less than straightforward. For example, public contributors need to be paid, typically through individual universities involved with ARC, all of which have their own individual systems, processes and requirements. This can mean researchers asking contributors to interact with a series of systems and staff entirely separate from the research and engagement activities themselves.

## Discussion

NIHR ARC EoE’s community geography place-based approach enabled a network of collaboration with partners from across charities, public sector organizations, health and social care and universities. Taking a place-based approach can help to prioritize health and social care needs of local communities and increased focus on working with communities that have been underserved by research. There were reports of the increased capacity for research, including skills developed for the researchers and partners in the communities, and finally, the relationships developed in communities enabled research to be shaped by patients, the public, service users, carers and communities, to ensure it focuses on areas most important to the people it impacts.

**Fig. 3:**
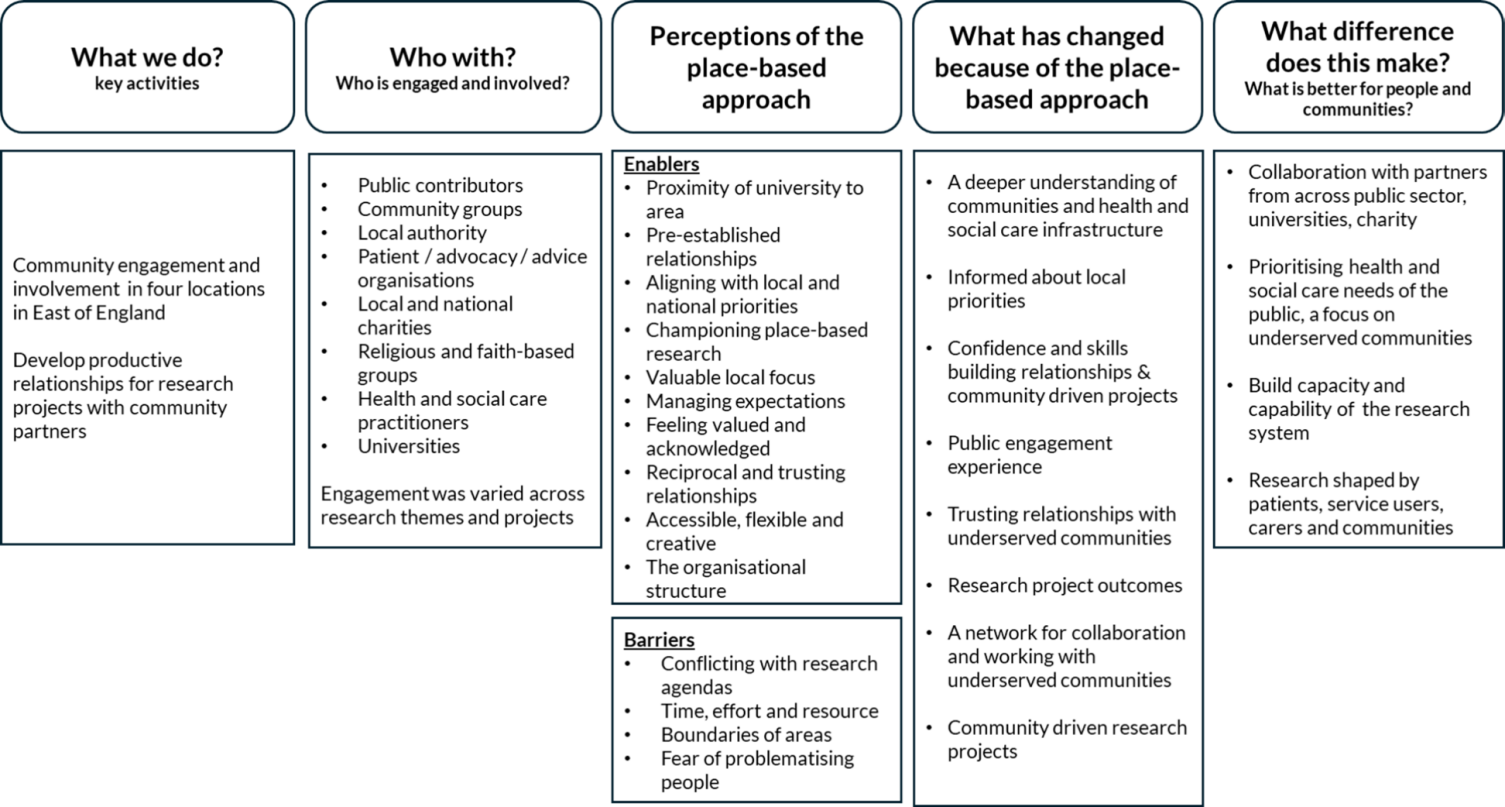
Adapted Research Contributions Framework of NIHR ARC EoE’s place-based approach

Figure 3 proposes a model exploring the contribution of the approach, applying the principles of a contribution analysis framework [8, 31]. The findings highlighted barriers to this approach that are presented in the model. These demonstrate that systemic barriers are not distinct to place-based approaches but consistently impact opportunities for engagement and involvement. In line with literature, our findings highlight the enablers that help to sustain relationships, such as the importance of reciprocal relationships, trust, feeling valued and acknowledged, accessible and flexible approaches, and having clear expectations [46–48]. There was also value in taking a place-based approach for research engagement as this aligned with principles and approaches that are ever present in the public health system [49]. The model presents learning, changes in knowledge, attitudes or skills developed, and changes to behaviour or practice and the potential overall difference made. This includes drawing out how the influence of engagement with communities through research infrastructure is incorporated into existing community knowledge, systems and experiences, such as through the development of productive networks for collaboration for research and for working with underserved communities, research that is shaped by communities and building research skills and experience across the system. Reference to the NIHR Outcomes Framework [50] has been made to map the important and aspirational, long-term outcomes of the approach (“What difference does this make?”), embedded within a regional research infrastructure.

### Contributions to the literature

Our findings support the added value of research infrastructure, beyond academic outputs, and the contribution approach as being helpful in considering infrastructure as a value-added platform for innovation. A strength of regional research infrastructure is its ability to connect across and account for complex and adaptive health, care, research and community systems and that it enables a systems-centric perspective [51, 52]. Coen et al (2010) argue for a relational conceptual framework for research infrastructures owing to the relationships between the interacting elements of infrastructure that define it. They also propose the role of internal research culture, identities and knowledge that are central to success (despite not being quantifiable) [36, 51]. Research infrastructure enables these individual relationships and the research community to be connected through a wider network and across the complex system. We also argue for the role of research infrastructures to be evaluated through a complex, system-level perspective, not only including but also building on societal, economic and academic outputs [8].

The last 20 years have seen an interdisciplinary so-called infrastructure turn, emerging from regional studies and disputing the notion of infrastructure as limited to the neutral and the material [53]. A regional perspective has increasingly been applied to urban infrastructure, examining geographical and political aspects [53]. Our paper goes some way to extending this regional perspective to research infrastructure. Specifically, the principle that infrastructure can serve as an institutional vehicle for academics, practitioners and policy makers to collaborate (in research) and use research evidence to inform policy and practice [54]. By using a community geography frame, we have theorized how research infrastructure can support regional, place-based agendas. The approach facilitates sustainable, reciprocal partnerships in addition to broadening collaborative knowledge production and shared power [45].

Applying the RCF to explore the impact of research infrastructures acknowledges the reality that working within complex systems with multiple and diverse stakeholders makes the attribution of impact vastly complicated. The model recognizes the complex, nonlinear interactions and contributions, steadily progressing over a long period of time. The research infrastructure assessed in this paper is one of several in the region that (although with varying remits) aims to work with similar, if not the same, underserved communities through involvement and engagement in research.

Our paper highlights the central role of research infrastructure-wide approaches in providing a platform for networking and collaboration. Improving equity in research engagement, involvement and participation is essential and purposeful, and intentional engagement with individuals and communities who have typically been underserved by research can help to break down barriers and rebuild trust [55–57]. As noted in our findings, the platform can support investment in the development of so-called research capacity among communities – increasing the education, training and support of community members to meaningfully contribute to be the research process [58]. Our findings align with previously identified strategies for research partnerships with communities and organizations. The strategies prioritize relationships, capacity building and support, communication and engagement in planning, conducting and dissemination of research [16]. Engagement with communities has an indirect impact through influencing and being incorporated into existing community knowledge, systems, understanding, beliefs and experiences [33]. Furthermore, our findings also align with the noted challenges of establishing and sustaining community geography projects. Namely, that of divergent goals, funding inconsistencies and insecure employment [35].

As has been noted, the impact of research infrastructures is difficult to measure because it would rely on counterfactuals (what would have happened if the infrastructure were not there) [8]. However, it is safe to say, certainly from the point of view of the participants, that key research projects and outputs with marginalized communities would not have been possible without the stable research infrastructure support and without the place-based approach that it enabled. This aligns with the community geography focus on producing knowledge that is action-focused and centred on engagement [35]. According to Shannon and colleagues [28], the key principles of community geography are: a focus on place and place-based concerns; diverse positionalities (across ethnic, classed, gendered and institutional boundaries); committed and reciprocal community partnerships; flexible epistemologies and methods; and open research practices and public scholarship. These principles are congruent with accounts of participants and the aims of the place-based approach, especially in terms of place and relationships. This paper adds to literature on research infrastructure by grounding its aims and rationale within ideas about social change and equity [59].

A defining characteristic of the results presented here is an emphasis on the need for strong relationships with partners in research. Typically, these take a good deal of time to develop, as explained by the participants, because communities may be hesitant to engage owing to several reasons, including mistrust in research and limited recognition of its benefits [60]. As has been outlined, establishing trust is a key factor in expanding the reach and relevance of research [57]. It is the relative stability and consistency of the NIHR ARC EoE research infrastructure that underpins these prolonged episodes of relationship building and provides the necessary resources.

### Strengths and limitations

To the best of our knowledge, this paper is the first contributions analysis framed review of a place-based approach to community partnership working at the research infrastructure level. We collected and compared responses from a range of researchers and community partners across the East of England region. As such, our findings have transferability to other regions in England and comparable infrastructures.

The researchers and community partners in the study were drawn from a necessarily limited sample of higher education institutions and disciplines. Added to which, the diversity and range of community organizations that make up the NIHR ARC EoE community partners could not be represented here. The authors held a dual role in being members of the research infrastructure and in undertaking this critical assessment, which may have influenced the interpretation of the data and reduced the critical distance from the topic. In addition, the study was qualitative, and future studies would also benefit from quantitative data collection, possibly by quantifying the perceived achievements that emerged from our qualitative analysis. The retrospective nature of the data collection inherently may result in inaccuracies and recollection biases. Future applications of the RCF approach would benefit from early planning and inclusion of anticipated theories of change (such as that presented in this paper) and the ongoing tracking, monitoring and collection of evidence throughout the duration of the research infrastructure funding, to support more rigorous and comprehensive interpretations of the influence of infrastructure over time [31].

## Conclusions

This paper critically examines the perceived impact of an approach to working with communities through research infrastructure, informed by both community geography principles and a contributions analysis framework. We therefore present the NIHR ARC EoE infrastructure as a community geography undertaking. The place-based approach was valued by participants and enabled opportunities to work with (rather than do to) communities that had previously been underserved by research and where the development of trusting relationships was key. The value of a place-based approach is widely applicable to any research infrastructure aiming to collaborate, involve and engage communities in research. However, the challenges of maintaining relationships and operationalizing collaboration against the backdrop of bureaucratic processes and competing agendas, serves to highlight the gap between the aims and ideologies engagement and the realities of the institutions they are implemented within. Research infrastructures are complex systems. As such, they have the potential to harness the power of connecting diverse communities, organizations and individuals [51, 52]. To do so, strategies to promote organizational–cultural change and dismantle infrastructural barriers are necessary [61].

## Data Availability

Data sets used and analysed during the study are not publicly available because they contain information that could compromise research participant consent and anonymity. Data sets are available from the corresponding author on reasonable request and subject to permission from NIHR ARC EoE.

## Abbreviations

NIHR: National Institute for Health and Care Research
ARC EoE: Applied Research Collaboration East of England
RCF: Research Contributions Framework
